# Association Between Covid-19 Severity And Residing In High Lead Level Locations.

**DOI:** 10.51894/001c.35880

**Published:** 2022-09-06

**Authors:** Vanessa Foxworth, Larry Kage, Kimberly Barber

**Affiliations:** 1 Family Medicine Ascension Genesys Hospital; 2 Clinical & Academic Research Ascension Genesys Hospital

**Keywords:** sars-CoV-2, covid-19, lead, health disparities, epidemiology

## Abstract

**INTRODUCTION:**

This 2021 retrospective study explored the association between patients that resided in high lead-exposed areas and Covid-19 severity.

**METHODS:**

Adults that resided within a metropolitan area hospitalized with Covid-19 at a community hospital between January 2020 and November 2020 were included in the study. Data including patient’s age, sex, length of stay, and co-morbid conditions were extracted from the hospital electronic health record. The patients were classified according to severity of disease based on a Covid Severity Index (qCSI) score, using patient’s vitals upon admission. Patient locations were classified per EPA mapping for lead exposure from water pipes.

**RESULTS:**

The qCSI score was significantly higher in the high exposure group, with a mean of 4.6 (SD = 4.4), than the low exposure group, which had a mean of 2.1 (SD = 3.2) (p = 0.004). The median risk stratification levels differed significantly (p = 0.006). Length of stay was also significantly greater in the high exposure group, mean 11.4 (SD 10.7), then in the low exposure group, mean 6.2 (SD = 7.2) (p = 0.01).

**CONCLUSION:**

This study demonstrated an association between Covid-19 severity and patients that have had high lead level exposure. Further research is needed to explore this possible association, such as studies involving larger datasets.

## INTRODUCTION

Lead exposure has been associated with poor health outcomes. Lead toxicity can lead to many acute, subacute, and even chronic symptoms. Symptoms of acute lead toxicity [i.e., greater than 45 microgram/dL(mcg/dL)] is nonspecific, including anemia, abdominal pain, fatigue, headache, and constipation.^1^

However, chronic low-level environmental exposure (low as 5-10 mcg/dL) is an often-overlooked risk factor for renal and cardiovascular disease. In one US cohort study, a blood lead level greater than 10 mcg/dL was associated with an increase in all-cause mortality as well as death due to cardiovascular diseases and cancers.^2^ The effect of lead toxicity on the body at the molecular level are mostly unknown and its effects on several systems are still being explored. However, one cause may be due to free radical damage, altering deoxyribonucleic acid (DNA) methylation.^3^

Another study has shown low-level lead exposure plays a role in telomere shortening and lipid disturbance, contributing to cardiovascular disease.^4^ Thus, lead exposure effects to the body are multi-factorial, leading to an overall detriment in health, a decrease in immune function, and an increase in risk factors related to poorer outcomes for disease.

Since several cities have declared federal emergencies for their water systems as of 2016, additional funding has been designated in response to the lead level crisis.^5,6^ For one such city, (Flint, MI), the local leaders chose to stop purchasing City of Detroit water and began pulling water from the local Flint River without implementing proper treatment. The resulting caustic water caused lead to be mobilized from within the city pipes, exposing citizens to lead for 18 months from April 2014 to October 2015.^7^

Since that period, the water supply has been switched back to the City of Detroit and as of March 2020, approximately 90% of the Flint city homes have been inspected and pipes replaced.^7,8^ However, past lead exposure can have lingering effects on the body. Ingested lead is absorbed into the bloodstream and has a serum half-life of approximately one month (the time required for the concentration of drug in the body to be reduced by one-half).^9^ Lead can then sequester from the bloodstream into other bones and organs.^9^ In adults, approximately 95% of lead sequesters to bones and teeth, while some lead can sequester to other organs such as liver, kidney, lungs, brain, and muscle.^10^

Most notably, lead can remain in cortical bone in an adult with a half-life between 10-30 years. Lead that sequesters in bone can slowly mobilize back into the bloodstream, increasing during states of malnutrition, physiological stress & illness, advanced age, hyperthyroidism, immobilization, kidney disease, and calcium deficiency.^9^ Thus during a body’s time of stress, lead that mobilizes back to the bloodstream can contribute to impaired immune function and multi-system organ damage for years.

Although the city of Flint responded to the water crisis, these residents continue to carry the dual burden during a pandemic of poor health and past lead exposures. Several risk factors have been linked to Covid-19 severity of illness including advanced age, cardiovascular disease, diabetes, chronic lung disease, cancers, chronic kidney disease, obesity, and smoking.^11^ A disproportionate burden of Covid-19 deaths among minorities has been demonstrated from the current pandemic. Non-Hispanic black patients account for 34% of Covid-19 deaths, despite making up only 13% of the US population.^12^ Thus, there is an increased risk of more severe illness among those with chronic illnesses and among minorities.

Although we now know that age, chronic disease, and obesity increases the risk of greater severity of illness from Covid-19, the additional risk of lead toxicity has not been explored for Covid-19. There has yet to be research demonstrating whether living in lead exposed areas increases the risk of greater Covid-19 severity. This study explored whether an association exists between lead exposure and Covid-19 severity.

## METHODS

This retrospective case/control study analyzed patients admitted to a 480-bed community teaching Hospital between Jan 1, 2020 and September 1, 2020 who had been diagnosed with Covid-19. This study was approved by the local IRB on November 10, 2020. Patients ages 18 or older who had a documented positive diagnosis for Covid-19 in the medical record were included.

**Figure 1.**
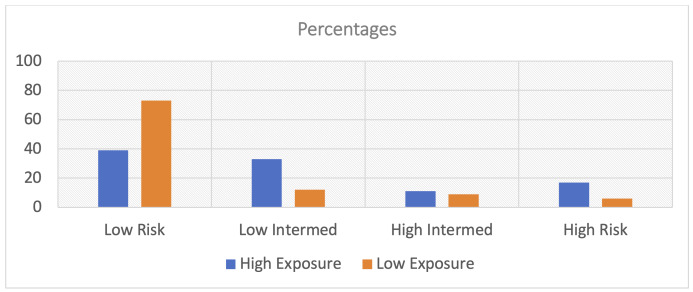
Covid-19 Severity Risk Stratum Breakdown by Exposure Group

The following data were extracted from the electronic health record: age, sex, date of admission, length of stay, if patient expired during hospitalization, and co-morbid conditions (history of pulmonary disease, cardiovascular disease, chronic kidney disease, diabetes, immunocompromised status), obesity, and smoking status. The patient was also classified according to severity of disease (outcome) based on the quick Covid Severity Index (qCSI) score (Figure 1), using patient’s vitals upon hospital admission.

The gCSI score is based on clinical variables with four levels (i.e., low, low intermediate, high intermediate, high) and the tool has been validated demonstrating a strong correlation coefficient of 0.81.^13^ Patients were then categorized into the four severity levels depending on their score (cases: high severity / controls: low or intermediate). A score of three or less indicated low likelihood 24-hour hour respiratory critical illness, with a mean outcome rate of 4% in the independent validation cohort. A score of four to six indicated low-intermediate risk with a risk of 24-hour critical illness at 30%.

A score of seven to nine indicated high-intermediate risk with a risk of 24-hour critical illness at 44%. A score of 10-12 indicated high risk with a risk of 24-hour critical illness at 57%. A risk of critical illness is defined by oxygen requirement >10 L/min by low-flow device, high-high flow device, non-invasive or invasive ventilation or death.

Figure 1. **gCSI Score Calculation**

*The quick COVID-19 Severity Index (qCSI: score range 0 to 12) is a prediction tool that includes 3 variables obtained from electronic health records ≤4 hours after patient presentation: respiratory rate, in breaths/min (≤22 = 0 points; 23 to 28 = 1 point; >28 = 2 points); lowest pulse oximetry (>92% = 0 points; 89% to 92% = 2 points; ≤88% = 5 points); and oxygen flow rate, in L/min (≤2 = 0 points; 3 to 4 = 4 points; 5 to 6 = 5 points). Lower scores indicate lower risk for respiratory decompensation at 24 hours. An online calculator is available (https://covidseverityindex.org/).

A score cut point of greater than 3 has very good diagnostic accuracy [Sensitivity: 79% (65 to 93), Specificity 78% (72 to 83), LR+ of 3.55, LR- of 0.27, and AUC: 0.81 (0.73 to 0.89)].^13^

Patients meeting all criteria had their location address abstracted to categorize each patient address into zones designated as having had high lead exposure or having no to low lead exposure from water pipes (exposure). The cut-off for having high lead exposure was greater than or equal to five ppb.

Environmental Protection Agency (EPA) mapping and list of addresses on lead exposure areas within the city and greater city areas from 2016 was used to designate potential exposure at the individual level found on the state public health website. Patient addresses were inventoried according to street locations within zip codes since 2016 and each patient was designated dichotomously into lead exposed zone or a non-lead exposed zone.

### Plan of Analysis

The primary exposure of interest was lead exposure based on address location measured dichotomously as a location of high lead exposure versus low and no lead exposure. The primary outcome of interest was Covid-19 severity based on the qSCI rating scale. Severity ratings categorized according to the quick Covid Severity Index (qCSI) as low (qCSI <3), Low-intermediate (4-6), high-intermediate (7-9) and high severity (10-12).

Chi square analysis was conducted to determine the overall association between lead exposure and Covid-19 severity. The Independent Student t-Test was used to compare total qSCI scores between severity subgroups and lead exposure groups.

To examine the association between lead and COVID-19 severity, we used multiple linear regression with two incremental levels of adjustment: Model 1 was unadjusted; Model 2 was adjusted for age, sex, obesity, oxygen saturation and co-morbid diseases (e.g., pulmonary disease, heart disease, and chronic kidney disease).

## RESULTS

A total of 129 patients were included in the study sample. The mean age of the total sample population was 61.7 years (SD = 17.3) with 63 (48.8%) males and 66 (51.2%) females. A total of 53 (41.1%) were obese and only 1 (0.8%) had reported smoking history. The overall hospital length of stay (LOS) was a mean of 6.96 (SD = 7.9) days with a range from 0 to 49 days. There were 28 (21.7%) with cardiovascular disease, 34 (26.4%) with diabetes, 17 (13.2%) with chronic kidney disease, and 1 (0.8%) with pulmonary disease. None of the study patients were immunocompromised.

The overall mean respiratory rate was 21.2 (SD = 8.9), mean pulse oxygen saturation was 93.2 (SD = 10.2), and the mean flow rate was 1.6 (SD = 2.4). The overall quick Covid-19 Severity Index (qCSI) score was 2.5 (SD = 3.5). The overall qCSI risk stratification was 88 (68.2%) low, 19 (14.7%) low-intermediate, 12 (9.3%) high-intermediate, and 10 (7.8%) high risk. No sample patients died during their hospitalization.

There were 18 (14%) patients in the high lead exposure group and 111 (86%) patients in the low or no exposure group. There was no difference in mean age between the high and low exposure groups, 63.9 (SD = 14.8) vs 61.4 (SD = 17.7), respectively, p = 0.57. Gender differed by group. There were 14 (77.8%) males in the high exposure group and 49 (44.1%) males in the low exposure group (p = 0.01). Additional patient characteristics are compared in Table 1. Pulmonary parameters did not significantly differ by lead exposure group (Table 1).

**Table 1.** Patient Characteristics by Exposure Group

**Factor**	**High Exposure**	**Low Exposure**	**p-value**
Age (mean, SD)	63.9 (14.8)	61.4 (17.7)	0.57
Gender (n, % Male)	14 (77.8)	49 (44.1)	0.01
Obesity (n, %)	6 (33.3)	47 (42.3)	0.47
Smoker (n, %)	0	1 (0.9)	--
Pulmonary Disease (n, %)	0	1 (0.9)	--
Cardiovascular Disease (n, %)	6 (33.3)	22 (19.8)	0.20
Chronic Kidney Disease (n, %)	3 (16.7)	14 (12.6)	0.63
Diabetes (n, %)	4 (22.2)	30 (27.0)	0.67
Immunocompromised (n, %)	0	0	--
Respiratory Rate (mean, SD)	22.2 (8.1)	21.1 (9.1)	0.63
Pulse Oxygenation (mean, SD)	89.3 (16.3)	93.8 (8.7)	0.10

The qCSI scores were significantly higher in the high exposure group, mean 4.6 (SD = 4.4) then the low exposure group, mean 2.1 (SD = 3.2), p = 0.004. The mean difference was 2.5 (CI: 1.5 - 3.5) which is a relative 54% difference. The median risk stratification levels differed significantly (p = 0.006) (Figure 1). There were 27.8% (n = 5) of the high exposure group scoring in the high risk of severity level versus 15.3% (n = 17) of the low exposure group, p = 0.004. The overall crosstabulation between exposure and severity showed that the high exposed group was significantly more likely to have been in the high severity group (27.8%) than the low severity group (15.3%) (relative difference 45%, p = 0.01).

The unadjusted regression analysis showed that lead was a significant predictor or Covid-19 severity (β=-2.43, CI: -4.1, -0.73; p = 0.006) The adjusted multiple regression analysis showed that lead exposure was a significant independent predictor of Covid-19 severity (β=-1.6, CI: -.02, -3.1; p = 0.04). Additional independent risk factors were presence of cardiovascular disease (β=1.6, CI: .31, 2.9; p = 0.02) and low oxygenation (β=-.14, CI: -.10, -.20; p < 0.01).

Length of stay was significantly greater in the high exposure group (mean 11.4, SD = 10.7) then in the low exposure group (mean 6.2, SD = 7.2), p = 0.01. The mean difference was 5.2 (CI: 3.61, 6.79) with a relative 45.6% difference. Lead was the only significant independent predictor of LOS (β= -4.8; CI: -.71, -8.9; p = 0.02) after adjusting for CVD, CKD, DM, age, gender, obesity, and oxygen saturation. None of these other factors in the model were significant.

## DISCUSSION

Even in the presence of chronic disease and low oxygenation, patients residing in lead exposed areas had a significantly increased risk of severe Covid-19 illness compared to those in non-exposed areas. Although the sample was not ideally powered, the magnitude of effect was large and statistically significant with a relative 54% increased risk in severity for the exposed group and good precision in the mean severity difference (4.6 vs 2.1 qCSI; p=0.004).

Since the sample included all consecutive patients during the study period who had been admitted and tested positive for Covid-19 at our institution, the study sample was representative of the hospital’s admissions during the pandemic.

This association was also confirmed in the length of stay (LOS) severity surrogate measure. The high lead exposed group had a 45.6% relative increase in LOS compared to the non-exposed group. The mean 5.2 difference in hospitalized days was statistically significant with good precision (p=0.01). After adjustment for the other clinical and patient characteristics, lead (was the only independent, significant predictor in the model (β=-1.6, CI: -.02, -3.1; p<0.01).

Lead exposure is linked to lasting poor health outcomes and when a pandemic occurs in and surrounding urban areas stricken by lead exposure, the consequences have implications for acute as well as chronic health.^14^ Although cities have been upgrading their water infrastructure since 2016, there continues to be delays in the removal of lead from drinking water pipes with corresponding delays in the return of health for residents exposed.^8^ This puts urban residents, already at risk of poor chronic health, at potential compounded risk from the negative outcomes inherent to infectious disease outbreaks.^15^

Furthermore, even if water infrastructures are fixed, residents can continue to have chronic poor health outcomes from past lead exposure.^14^ This shows the danger of building poor water infrastructure in the first place and should place an increase importance of implicating water safety bills and legislation.

### Study Limitations

One limitation of the study is that lead exposure was identified at the individual level rather than at the regional level. Participants were also selected based first on hospitalization and then linked based on household location of lead exposure. This method turned out to be precise but may have missed several residents hospitalized elsewhere or who had moved in or out of the area recently.

Another limitation is the differentially small sample size. Although it was powered for the primary and secondary outcomes, the lack of significance in the adjusted variables of the regression model may differ in larger study.^14^ We acknowledge that we could not confirm whether the patients would have remained in the same address and zip code at the time of the 2014 Flint water crisis and the past year when they were admitted to the hospital for Covid-19. It is the assumption that the patients in this study would not have changed addresses.

Furthermore, lead absorption into the body can differ between individuals, our results may be weaker than what larger studies can demonstrate.^14^ Further investigation are needed to explore the link between poor nutrition, lead absorption, and poor Covid-19 health outcomes with future studies.

## CONCLUSION

Based on these study results, a potential association between lead exposure and viral infections should be taken into consideration before the next pandemic occurs. Vulnerable patients who are also lead exposed, may need greater attention and increased screening to provide the targeted treatment necessary to overcome their health deficits worsened by toxicity. Information generated from this study may enhance recognition of the importance of lead level screening among viral, respiratory infected patients in exposed areas.

### Conflicts of Interest

None
